# Constitutive PGC-1α overexpression in skeletal muscle does not protect from age-dependent decline in neurogenesis

**DOI:** 10.1038/s41598-019-48795-w

**Published:** 2019-08-23

**Authors:** Lars Karlsson, María Nazareth González-Alvarado, Reza Motalleb, Klas Blomgren, Mats Börjesson, Hans Georg Kuhn

**Affiliations:** 1 Institute for Neuroscience and Physiology, Center for Brain Repair and Rehabilitation, University of Gothenburg, Box 436, 405 30 Gothenburg, Sweden; 2 The Queen Silvia Children’s Hospital, Sahlgrenska University Hospital, 416 85 Gothenburg, Sweden; 3 Department of Women’s and Children’s Health, Karolinska Institutet, 171 64 Stockholm, Sweden; 4 Pediatric Oncology, Karolinska University Hospital, 171 64 Stockholm, Sweden; 5 Center for Health and Performance, Department of Food and Nutrition, University of Gothenburg & Department of Neuroscience and Physiology, Sahlgrenska Academy, Box 115, 405 30 Gothenburg, Sweden; 6 Sahlgrenska University Hospital/Östra, 416 50 Gothenburg, Sweden

**Keywords:** Senescence, Mechanism of action, Animal breeding, Adult neurogenesis, Neurotrophic factors

## Abstract

Aerobic exercise prevents age-dependent decline in cognition and hippocampal neurogenesis. The transcription factor peroxisome proliferator-activated receptor gamma co-activator 1-alpha (PGC-1α) mediates many of the exercise-induced benefits in skeletal muscle, including the release of factors into the circulation with neurotrophic effects. We use a transgenic mouse model with muscle-specific overexpression of PGC-1α to study the contribution of chronic muscle activation on exercise-induced effects on hippocampal neurogenesis in aging. Young and old transgenic and wild type animals of both sexes displayed a robust age-related reduction in newborn BrdU^+^-cells, immature neurons (DCX^+^-cells) and new mature BrdU^+^/NeuN^+^-neurons in the dentate gyrus. No differences were detected between genotypes or sexes. Analysis of serum proteins showed a tendency towards increased levels of myokines and reduced levels of pro-inflammatory cytokines for transgenic animals, but only musclin was found to be significantly up-regulated in transgenic animals. We conclude that constitutive muscular overexpression of PGC-1α, despite potent systemic changes, is insufficient for mimicking exercise-induced effects on hippocampal neurogenesis in aging. Continued studies are required to investigate the complex molecular mechanisms by which circulating signals could mediate exercise-induced effects on the central nervous system in disease and aging, with the aim of discovering new therapeutic possibilities for patients.

## Introduction

Regular aerobic exercise, or endurance exercise, is a potent therapeutic treatment that apart from increasing well-being and general health, also reduces the risk and treats a variety of chronic conditions in the body and brain, such as cardiovascular, metabolic, autoimmune, cerebrovascular, psychiatric, neurodegenerative diseases, and many others^1^. Endurance exercise also improves cognition, especially during aging, by enhancing several aspects of neuroplasticity^2–4^. These effects are partly a result of increased blood flow and neurotrophic signaling in the brain, most prominently brain-derived neurotrophic factor (BDNF), which mediates enhanced neurogenesis, synaptic plasticity, and angiogenesis in the dentate gyrus (DG) of the hippocampus^4^. However, the contributions of different mechanisms mediating exercise-induced effects in the body and brain are still unclear, especially the essential involvement of signals in the circulation on the central nervous system (CNS)^5^. Through detailed studies of how molecular pathways contribute to exercise-induced effects, we are able to improve our understanding of regenerative possibilities for injured or degenerative tissue, as well as to optimize physical therapy for different conditions. Due to physical^6^ or mental^7^ constraints, whether induced by diseases or genetic makeup^8^, many patients are unable to get the full benefits of exercise, which calls for a search for possible pharmacological targets that could confer some aspect of exercise-related effects on the brain.

Skeletal muscle undergoes adaptations from regular aerobic exercise that lead to improved mitochondrial density, oxidative capacity, glucose uptake, lipid oxidation, substrate utilization, angiogenesis, and muscle fiber-type switching^9,10^. The transcription factor peroxisome proliferator-activated receptor gamma co-activator 1-alpha (PGC-1α) is a regulator of mitochondrial biogenesis and plays a central role in adaptations to endurance exercise in skeletal muscle. PGC-1α mediates these adaptations through interactions with many transcription factors such as peroxisome proliferator-activated receptor-alpha (PPAR-alpha) and -delta (PPAR-delta), nuclear respiratory factor-1 and -2 (NRF1/2), estrogen-related receptor alpha (ERRα) and myocyte enhancer factor-2 (MEF2)^11^. Skeletal muscle is a well-documented endocrine organ, where activation of the PGC-1α pathway induces the release of exercise-induced myokines into the circulation, some of which are known to have potent effect on the central nervous system^5^. For example, fibronectin type III domain-containing protein 5 (FNDC5) is a messenger molecule released from PGC-1α induction in skeletal muscle capable of upregulating hippocampal BDNF levels^12^, indicative of a direct muscle-brain crosstalk.

Through upregulation of PGC-1α under the muscle creatinine kinase promoter in transgenic mice (MCK-PGC-1α), we can study the specific influence of activation of the muscular PGC-1α pathway on exercise-induced changes in the brain^9^. MCK-PGC-1α mice have a constitutively developed endurance muscle phenotype^9^, with protection from denervation- and disuse-induced atrophy^13,14^, as well as Duchenne muscular atrophy^15,16^. PGC-1α expression in skeletal muscle has been reported to be decreased with aging in both rodents and humans^17,18^, and MCK-PGC-1α animals are protected from age-related motor dysfunction^19,20^, with improved muscle mitochondrial function with aging and a gene expression profile similar to young mice^20,21^. Systemically, MCK-PGC-1α animals have an activity-dependent improvement in glucose homeostasis and insulin resistance^22^, improved kidney energy metabolism with protection from tubular damage^23^, and display protection from stress-induced neuroinflammation^24^. Further, PGC-1α is known to modulate many of the processes of aging, such as inflammatory profile, telomere dysfunction, mitochondrial dysfunction, oxidative stress, insulin resistance, and genomic instability^25^. MCK-PGC-1α animals display an increased life span^21^ and age-dependent telomere dysfunction causes a repression of PGC-1α expression with mitochondrial defects that can be rescued by PGC-1α overexpression^26^.

Adult hippocampal neurogenesis occurs throughout life, but gradually diminishes during aging, a deterioration process considered to contribute to age-dependent cognitive decline^27,28^. This decline in neurogenesis during aging can be ameliorated by different means including endurance exercise^29^. To determine if chronic activation of skeletal muscle along with improved muscle metabolism could prevent age-dependent decline in neurogenesis, we study hippocampal neurogenesis in young and middle-aged mice with overexpression of the transcription factor PGC-1α in skeletal muscle. Further, previous reports in humans have suggested that there are sex differences in skeletal muscle composition and expression patterns, including differences in PGC-1α expression^30^. To elucidate if chronic muscle activation could influence sex-dependent effects on hippocampal neurogenesis, we study both middle-aged female and male mice.

## Methods

### Animals

Transgenic MCK-PGC-1α animals on C57BL/6J background (The Jackson Laboratory; Stock no. 008231), a kind gift from Dr. Bruce Spiegelman (Harvard Medical School, Boston, MA), have been previously described^9^. Housing and breeding conditions have been described previously^31^. All experiments were approved by the Gothenburg ethical committee on animal research (#317-2012 and #181-2015) and were performed in accordance with relevant guidelines and regulations. Genotyping was performed as described previously^31^.

### BrdU Labeling

Group-housed male and female wildtype (WT) and transgenic (TG) mice were given daily intraperitoneal injections of BrdU (50 mg/kg) for 5 consecutive days at 2 and 10 months of age. Fifteen female (WT, 8; TG, 7) and 25 male (WT, 8; TG, 17) 3-month-old animals, and 22 female (WT, 17; TG, 8) and 21 male (WT, 9; TG, 12) 11-month-old animals, were included in the experiments. Animals were euthanized and perfused 28 days after the first day of BrdU injection for immunohistochemical analysis of cytogenesis and neurogenesis. In the 11-month-old female WT group one animal died during injections.

### RT-qPCR

Male mice at 8 months of age were decapitated under isoflurane anesthesia. Hippocampi, pre-frontal cortex, and gastrocnemius muscle, were dissected and snap-frozen in isopentane containing dry ice. RNeasy Lipid Tissue Kit and RNeasy Fibrous Tissue Kit (Qiagen, Hilden, Germany) were used to extract RNA from brain and muscle tissue, respectively. First strand DNA synthesis and analyses of RNA integrity and purity was performed as previously described^31^. Quantitative PCR was performed according to MIQE guidelines^32^ as previously described^31^. Primer pairs used for qPCR can be found in Supplementary Table S1.

### Tissue processing

Four weeks following start of BrdU injections, mice were deeply anesthetized with a peritoneal injection of 50 mg/kg sodium thiopental during the inactive phase of the animals. Blood was extracted through cardiac puncture using a 27-gauge needle, which was allowed to coagulate in a low protein binding microcentrifuge tube (Maxymum Recovery, Corning Life Sciences, Corning, NY, USA) for 1 h. After centrifugation at 3,000 *g* for 10 minutes, serum was transferred into a new low protein binding tube, frozen on dry ice and stored at −80 °C until further use. Animals were transcardially perfusion-fixated and brains were subsequently immersion-fixated, using paraformaldehyde, as previously described^31^. Brains were sectioned and stored as previously described^31^. In the 3-month-old male transgenic group, two animals were not sectioned for immunohistochemical analysis.

### Immunohistochemistry

Free-floating immunohistochemistry was performed as previously described^31^. Primary antibodies were diluted in blocking solution and incubated at 4 °C for 3 days in goat anti-DCX (1:250, sc-8066, Santa Cruz Biotechnology, Dallas, TX, USA) or 2 days in rat anti-BrdU (1:500, OBT0030, AbD Serotec, Kidlington, UK) and mouse anti-NeuN (1:2000, MAB377, Millipore, Burlington, MA, USA). After rinsing, sections were incubated at RT for either 1 hour (biotinylated) or 2 hours (fluorescence) with secondary antibody in blocking solution as follows: with biotinylated donkey anti-goat (1:2000, 705065147, Jackson ImmunoResearch Laboratories, Cambridge, UK) for DCX-DAB, and with donkey anti-mouse 555 IgG (1:1000, A21202, Molecular Probes, Eugene, OR, USA) and donkey anti-rat 488 IgG (1:1000, Molecular Probes, A21208) for BrdU/NeuN immunofluorescence. For immunofluorescence, sections were rinsed and mounted from 0.1 M PBS onto glass slides. After allowing sections to dry, glass slides were submerged in 1% Sudan Black in EtOH for 5 minutes and rinsed thoroughly in PBS before coverslipping.

For analysis of BrdU^+^-cells and BrdU^+^/NeuN^+^-cells in 3-month-old animals, 8 female WT, 8 female TG, 7 male WT, and 12 male TG were stained and quantified (1 female WT and 1 male TG was excluded from analysis due to insufficient number of sections after mounting, and 1 female WT and 1 male TG were excluded from analysis due to mixing of sections during staining). For analysis of DCX^+^-cells in 3-month-old animals, 8 female WT, 7 female TG, 8 male WT, and 12 male TG were stained and quantified (1 female TG and 2 male TG were excluded from analysis due to insufficient number of sections after mounting). For volume analysis of different sub-regions of the DG in 11-month-old animals, 10 female WT, 8 female TG, 9 male WT, and 11 male TG were stained and quantified (1 animal in male TG was excluded from analysis due to insufficient number of sections after mounting). For analysis of BrdU^+^- and BrdU^+^/NeuN^+^-cells in 11-month-old animals, 14 female WT, and 8 female TG, 9 male WT, and 12 male TG were stained and quantified. For analysis of DCX^+^-cells in 11-month-old animals 9 female WT, 7 female TG, 9 male WT, and 9 male TG were stained and quantified.

### Imaging and quantification

Investigator-blinded stereological quantification was performed as previously described^31^. For analysis of NeuN/BrdU co-labeling, a LSM 700 confocal microscope was used (Carl Zeiss AG, Oberkochen, Germany). Co-localization was determined at 20× optical magnification at close to 1 airy-units (AU) with 2.5× digital zoom and a sequential scanning mode. Image processing was performed as previously described^31^.

### Multiplex protein analysis

Serum from 11-month-old male animals was thawed and applied to multiplex assays for detection of serum chemokines, cytokines (ProcartaPlex, EPX360-26092-901, Thermo Fisher Scientific) and myokines (Milliplex. MMYOMAG-74K, Merck, Kenilworth, NJ, USA). Multiplex microplates were analysed using a Bio-Plex 100 system (Bio-Rad) according to manufacturers’ instructions. Concentrations were determined based on the average of technical duplicates and wells with bead count of less than 20 were excluded for high precision. In cases where the concentrations were below the detection range, the concentration was set to the minimum detection limit.

### Statistical analysis

Data were processed and analysed using Microsoft Excel 2017 (Microsoft Corp.) and GraphPad Prism 8 (GraphPad Software). Quantitative PCR data were analysed as previously described^31^. Appropriate tests were selected, as specified in the text, based on normality and homogeneity of variance. Normality was determined by visual inspection of density plotted logged and unlogged data. For data adhering to normality and equality of variances, two-way analysis of variance (ANOVA) was used. Protein concentration data was evaluated two-tailed with t-test for normally distributed analyte data and Mann Whitney test for non-normally distributed analyte data. All analyte data was treated systematically without discrimination, and false discovery rate (FDR) was calculated with the Benjamini-Hochberg method using an online calculator^33^. We used a significance level of 0.05 for all test, except for the rigorous FDR adjustments where we selected a significance level of 0.1.

## Results

Mice with muscle-specific overexpression of PGC-1α in skeletal muscle^9^ were used to investigate if animals with an endurance exercise muscle phenotype would show enhanced neurogenesis. In particular, we studied effects of genotype, aging, and sex, on hippocampal neurogenesis.

### Validation of PGC-1α Overexpression in skeletal muscle

**Figure 1:**
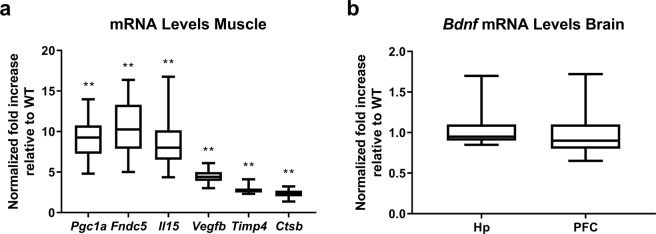
Validation of PGC-1α overexpression in skeletal muscle. Graph (**a**) shows relative mRNA levels of *Pgc1a*, fibronectin type III domain-containing protein 5 (*Fndc5*), interleukin 15 (*Il15*), vascular endothelial growth factor B (*Vegfb*), metalloproteinase inhibitor 4 (*Timp4*), and cathepsin B (*Ctsb*), normalized to reference genes *18S* and *Gapdh* (Pfaffl method, resampling test). Image (**b**) shows relative mRNA levels of *Bdnf* in hippocampus and PFC, normalized to reference genes *18S*, *Actb* and *Tbp* (Pfaffl method, resampling test; n.s.). Data expressed as median, with interquartile range as box and minimum and maximum values as whiskers for WT and MCK-PGC-1α animals (n = 5–6; **p < 0.01). *Hp*, *hippocampus*. *PFC*, *pre-frontal cortex*.

Transgenic mice displayed upregulated mRNA levels of *Pgc1a* along with downstream effector genes, *Fndc5*, *Il15*, *Vegfb*, *Timp4* and *Ctsb* (Fig. 1a). No difference in *Bdnf* gene expression existed between genotypes in hippocampus or pre-frontal cortex (Fig. 1b).

### Skeletal muscle PGC-1α overexpression and age-dependent decline in neurogenesis

**Figure 2:**
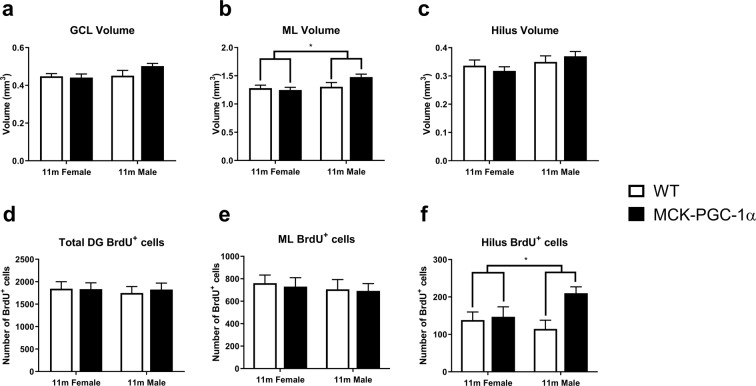
Overexpression of PGC-1α in skeletal muscle does not influence hippocampal volume or cytogenesis in middle-aged animals. Graphs showing volumes of DG subregions (**a**) GCL (two-way ANOVA; n = 8–10; n.s.), (**b**) ML (two-way ANOVA, n = 8–10; sex effect, *p < 0.05; genotype and interaction effect, n.s.), and (**c**) hilus (two-way ANOVA; n = 8–10; n.s.) in 11-month-old female WT and transgenic animals. Graphs showing corresponding number of BrdU^+^ cells in the (**d**) DG, (**e**) ML and (**f**) hilus (two-way ANOVA; n = 9–14; genotype effect, *p < 0.05) 4 weeks after the first BrdU-injection. Data expressed as mean ± standard error of the mean (SEM) for WT and MCK-PGC-1α animals.

Using young and middle-aged animals, we investigated the potential longitudinal protective effects from chronic muscular overexpression of PGC-1α on hippocampal neurogenesis in aging along with possible differences between sexes. Group-housed female and male animals were injected with BrdU (50 mg/kg i.p.) during 5 days and analysed 4 weeks later. For male and female MCK-PGC-1α animals at 11 months of age, the subregional volumes of the DG, i.e. the granular cell layer (GCL), molecular layer (ML) and hilus, showed no significant differences between genotypes or sexes in the GCL or hilus (two-way ANOVA, n = 8–10; main and interaction effects, n.s.), with the exception of a larger volume of the ML in male animals (two-way ANOVA, n = 8–10; sex effect, F(1, 30) = 4.6, p = 0.04; genotype and interaction effect, n.s.; See Fig. 2). For 11-month-old females and males, the number of newborn cells in the DG subregions, we found no significant differences between genotypes or sexes in the GCL or ML (two-way ANOVA, n = 9–14; main and interaction effects, n.s.) with the exception of a higher number of BrdU^+^ cells in the hilus for transgenic animals (two-way ANOVA, n = 9–14; genotype effect, F(1, 39) = 5.4, p = 0.026; sex and interaction effect, n.s.; See Fig. 2). For full two-way ANOVA results see Supplementary Table S2.

**Figure 3:**
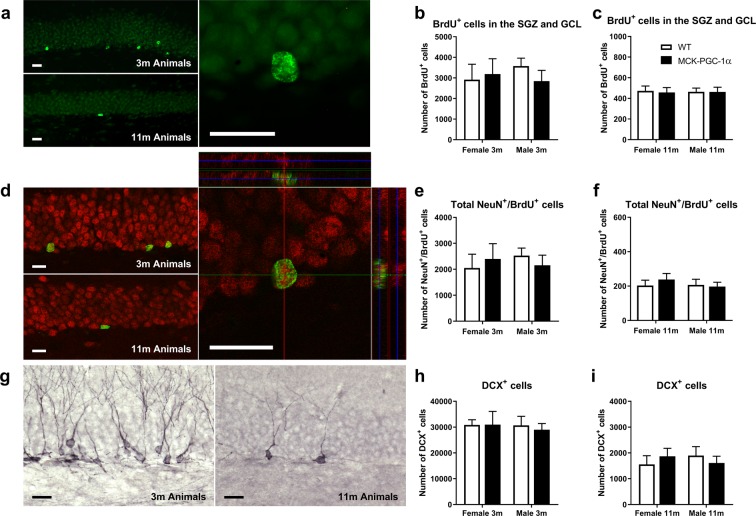
Overexpression of PGC-1α in skeletal muscle does not protect against reduction of hippocampal neurogenesis in aging. (**a**) Images showing BrdU immunostainings of DG for 3- and 11-month-old animals 4 weeks after first BrdU-injection, with corresponding graphs representing number of BrdU^+^-cells in the SGZ and GCL for (**b**) 3- (two-way ANOVA; n = 6–10; n.s.) and (**c**) 11-month-old animals (two-way ANOVA; n = 9–14; n.s.). (**d**) Images showing NeuN/BrdU immunostainings of DG for 3- and 11-month-old animals with confocal picture from quantification of NeuN^+^/BrdU^+^-cells with corresponding graphs representing number of NeuN^+^/BrdU^+^-cells for (**e**) 3- (two-way ANOVA; n = 6–10; n.s.) and (**f**) 11-month-old animals (two-way ANOVA; n = 9–14; n.s.). Images showing (**g**) DCX immunostainings of DG for 3- and 11-month-old animals with corresponding graphs showing number of DCX^+^ cells for (**h**) 3- (two-way ANOVA; n = 6–10; n.s.) and (**i**) 11-month-old animals (two-way ANOVA; n = 7–9; n.s.). Data expressed as mean ± SEM for female and male WT and MCK-PGC-1α animals. Scale bars = 20 µm.

**Figure 4:**
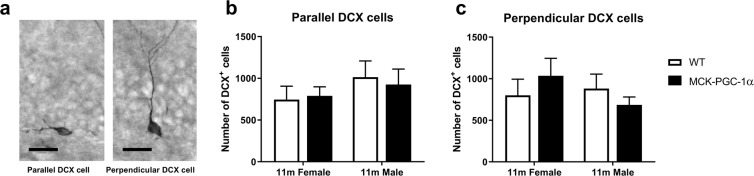
Overexpression of PGC-1α in skeletal muscle does not influence number of immature or mature DCX^+^-cells in the DG of middle-aged animals. Image showing DCX^+^ immunostainings of DG for 11-month-old animals demonstrating (**a**) parallel and perpendicular orientation, with corresponding graphs showing number of DCX^+^ cells with (**b**) parallel and (**c**) perpendicular orientation (two-way ANOVA; n = 7–9; n.s.) for 11-month-old animals. Data expressed as mean ± SEM for female and male WT and MCK-PGC-1α animals. Scale bars = 50 µm.

Further, we analysed the number of newly generated BrdU^+^-cells in the neurogenic region of the DG, consisting of the subgranular zone (SGZ) and the GCL, with respect to genotype or sex using 2-way ANOVA for 3- and 11-month-old animals (See Fig. 3a–c and Table 1). The number of BrdU^+^-cells was dramatically decreased with aging in both WT and transgenic animals of both sexes (See Table 2), but no difference existed between genotypes or sexes (Table 1). Similarly, the number of newly generated mature neurons determined by NeuN^+^/BrdU^+^ co-labeling decreased with aging without any differences between either genotype or sex for 3- and 11-month-old animals (See Fig. 3d–f and Tables 1 and 2). Numbers of immature neurons (DCX^+^-cells) were reduced with aging for both genotypes and sexes (See Fig. 3g–i and Table 2), but there were no significant differences for 3- and 11-month-old animals with respect to genotype or sex (See Table 1). Finally, we also analysed DCX^+^-cells in 11-month-old animals based on the classification by Winner and colleagues^34^. DCX^+^-cells with processes in parallel or perpendicular orientation with respect to the GCL border, indicate a more immature or more mature neuronal developmental stage, respectively. By using two-way ANOVA, we found no difference in numbers of DCX^+^-cells with parallel or perpendicular orientation between genotypes and sexes (two-way ANOVA, n = 7–9, main and interaction effects, n.s.; See Fig. 4). For full two-way ANOVA results see Supplementary Table S3.

**Table 1:** Genotype, sex and interaction effects of two-way ANOVA on number of newborn cells (BrdU), newborn mature neurons (NeuN/BrdU) and immature neurons (DCX) in the DG.

Analysis^a^	Genotype effect	Sex effect	Interaction effect
GCL BrdU^+^ cells 3 m^b^	0.15 (p = 0.71)	0.071 (p = 0.79)	0.70 (p = 0.41)
GCL BrdU^+^ cells 11 m^c^	0.036 (p = 0.85)	0.002 (p = 0.96)	0.02 (p = 0.88)
Total NeuN^+^/BrdU^+^ 3 m^b^	0.001 (p = 0.98)	0.06 (p = 0.80)	0.62 (p = 0.44)
Total NeuN^+^/BrdU^+^ 11 m^c^	0.16 (p = 0.69)	0.35 (p = 0.56)	0.48 (p = 0.49)
DCX^+^ cells 3 m^d^	0.06 (p = 0.81)	0.11 (p = 0.74)	0.077 (p = 0.78)
DCX^+^ cells 11 m^e^	0.002 (p = 0.97)	0.017 (p = 0.90)	0.86 (p = 0.36)

^a^See Fig. 3.

^b^Data presented as test value of F(1, 27) along with the corresponding p-value in parenthesis. ^c^Data presented as test value of F(1, 39) along with the corresponding p-value in parenthesis. ^d^Data presented as test value of F(1, 28) along with the corresponding p-value in parenthesis. ^e^ Data presented as test value of F(1, 30) along with the corresponding p-value in parenthesis.

**Table 2:** Genotype, age and interaction effects of two-way ANOVA on number of newborn cells (BrdU), newborn mature neurons (NeuN/BrdU) and immature neurons (DCX) in the DG.

Analysis^a^	Genotype effect	Age effect	Interaction effect
GCL BrdU^+^ cells males^b^	1.36 (p = 0.25)	75.3 (p < 0.0001)	1.34 (p = 0.25)
GCL BrdU^+^ cells females^c^	0.10 (p = 0.75)	43.4 (p < 0.0001)	0.14 (p = 0.71)
Total NeuN^+^/BrdU^+^ male^b^	0.65 (p = 0.43)	81.2 (p < 0.0001)	0.59 (p = 0.45)
Total NeuN^+^/BrdU^+^ females^c^	0.40 (p = 0.53)	45.0 (p < 0.0001)	0.27 (p = 0.61)
DCX^+^ cells males^b^	2.0 (p = 0.17)	90.8 (p < 0.0001)	0.61 (p = 0.44)
DCX^+^ cells females^c^	0.008 (p = 0.93)	158 (p < 0.0001)	0.002 (p = 0.96)

^a^See Fig. 3.

^b^Data presented as test value of F(1, 35) along with the corresponding p-value in parenthesis.

^c^Data presented as test value of F(1, 32) along with the corresponding p-value in parenthesis.

### Skeletal muscle PGC-1α overexpression and serum protein profile

**Figure 5:**
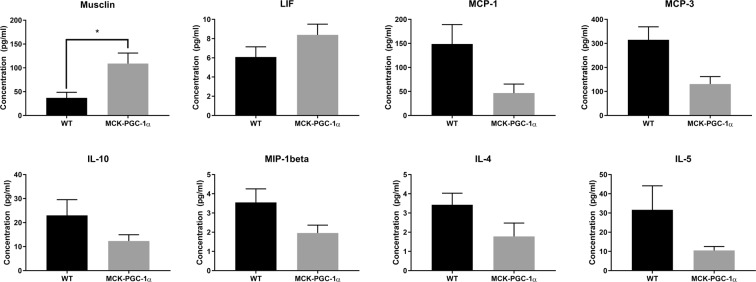
Changes in levels of circulating cytokines and myokines with muscle-specific overexpression of PGC-1α in middle-aged animals. Graphs showing protein concentrations of analytes with a statistical difference or a tendency toward a difference between genotypes (t-test; *p < 0.1; n = 7–10). See Table 3 for statistical analysis.

To investigate possible differences in serum proteins associated with overexpression of PGC-1α in skeletal muscle with aging, we extracted blood from 11-month-old male animals for concentration measurements of cytokines, chemokines, and myokines in serum. Analysis reveals that levels of monocyte chemoattractant protein-1 (MCP-1) and MCP-3, IL-4, IL-5 and IL-10, and macrophage inflammatory protein-1beta (MIP-1beta), were downregulated, and musclin and leukemia inhibitory factor (LIF), were upregulated in transgenic animals compared to WT (See Fig. 5 and Table 3). The entire list of analysed myokines can be found in the Supplementary Table S4. However, after FDR adjustment only musclin was significantly upregulated (Mann-Whitney; n = 7–10; p = 0.0029, FDR-adjusted p = 0.084) at a more than 2-fold higher concentration in transgenic compared to WT serum. Studying the relationship between serum protein levels and number of DCX cells, we found that eotaxin (chemokine ligand 11; CCL11) had a positive correlation between serum levels of the chemokine in WT, but not in transgenic animals (linear correlation; WT: r = 0.99, p = 0.01, n = 4; TG: r = 0.46, p = 0.35, n = 6).

**Table 3:** List of selected cytokines and myokines from analysis with parametric and non-parametric independent sample tests.

Analysis^a^	p-value	FDR-adjusted p-value
Musclin^b^	0.0029	0.084
MCP-1^c^	0.04	0.39
MCP-3^c^	0.01	0.15
LIF^b^	0.11	0.53
IL-10^b^	0.16	0.66
MIP-1beta^c^	0.08	0.58
IL-4^c^	0.1	0.58
IL-5^b^	0.16	0.58

^a^See Fig. 5.

^b^Mann-Whitney, n = 7–10.

^c^t-test, n = 7–10.

## Discussion

In this study, we used young and old, female and male transgenic mice with overexpression of PGC-1α under the control of muscle creatinine kinase promoter in order to determine the contribution of skeletal muscle PGC-1α signaling pathway in exercise-induced effects on brain plasticity and protection from age-dependent reduction in neurogenesis. The main result of the present study is that muscular overexpression of PGC-1α does not exert beneficial effects on adult neurogenesis, independent of sex or age, indicating that PGC-1α overexpression in muscle is not sufficient to phenocopy exercise-induced effects in aging.

We validated the transgenic model in terms of upregulated gene expression of *Pgc1a* and downstream effector genes in skeletal muscle and found mRNA levels comparable to previous studies using these transgenic mice^10,24,31^. In a prior study, we also confirmed that the transgenic mice developed an appropriate muscle phenotype with higher mitochondrial density by observing increased copy number ratio of mitochondrial to nuclear DNA^31^, a reliable measure of mitochondrial biogenesis and mass. While PGC-1α expression is induced in skeletal muscle and brain of exercising mice^12,35^, MCK-PGC-1α transgenic mice have chronic overexpression of PGC-1α in skeletal muscle, but no upregulation in the brain^19^. The moderate overexpression of PGC-1α in this mouse model is comparable to levels achieved by exercise^9,36^.

We determined that MCK-PGC-1α mice have increased muscular *Fndc5*, *Vegfb*, *Il15*, and *Timp4* mRNA levels. In a previous study, the protein product of these transcripts have been identified as likely to be secreted from PGC-1α overexpressing muscle^10^. Middle-aged MCK-PGC-1α animals appear to have similar *Pgc1a* mRNA levels, but relatively higher levels of *Fndc5*, *Il15* and *Vegfb* compared to young animals^31^. We also found cathepsin B (*Ctsb*) mRNA to be upregulated. These five factors, upregulated in muscle of MCK-PGC-1α mice, have all been associated with promoting neurogenesis. FNDC5 is a protein downstream of PGC-1α that is produced, cleaved and released into the circulation by skeletal muscle^10^. A forced peripheral overexpression of FNDC5 was reported to increase BDNF expression in the hippocampus^12^, which suggests a direct cross-talk between exercised skeletal muscle, hippocampal BDNF expression, and neurogenesis. From our studies we see that the chronic skeletal muscle activation through PGC-1α does not yield exercise-induced effects on neurogenesis. We have not determined protein levels of pro-BDNF and mature BDNF, and can therefore not exclude possible differences in post-translational modifications of this protein.

PGC-1α has been reported to induce VEGF which regulates angiogenesis and vasodilatation in skeletal muscle^10^, and levels of VEGF in muscle are also known to decline with age^37^. VEGF is considered to be released into the circulation from muscle during exercise, but also to be directly induced in the hippocampus following exercise, where it acts to promote angiogenesis, BDNF expression, synaptic plasticity and neurogenesis^38,39^. Interestingly, Rich and colleagues show that skeletal muscle VEGF is essential for exercise-induced neurogenesis^40^. Transgenic animals with muscle-specific overexpression of PGC-1α displayed increased levels of VEGF mRNA in muscle tissue. Since VEGF is released into the circulation following exercise and has been identified as the most important pro-angiogenic factor in most tissue^41^, the increased expression in skeletal muscle overexpressing PGC-1α may affect the microvascular system in the CNS. However, we did not observe a difference in vascular density in the neocortex between genotypes in a previous study^31^.

IL-15 is a pro-inflammatory cytokine regulating T- and natural killer cells and playing a role in fat metabolism and muscular hypertrophy^41^. The cytokine is produced by skeletal muscle during exercise, is capable of promoting neurogenesis^42^, and is reduced with aging^43^.

TIMP4, belonging to the tissue inhibitor of metalloproteinase, is induced in human skeletal muscle upon exercise and regulates muscle regeneration^10^. Even though the enzyme is rather uncharacterized, another member from the same family of enzymes, TIMP2, has been identified as an anti-aging factor with an important role in regulating hippocampal and cognitive function^44^. Finally, cathepsin B is a recently identified exercise-induced myokine released into the circulation that is associated with memory function and neurogenesis, first discovered to be secreted from 5-Aminoimidazole-4-carboxamide riboside (AICAR) treated muscle cells^45^.

Aging has a strong effect on neuroplasticity mechanisms, in particular on hippocampal neurogenesis, which is downregulated in rodents to very low levels in the aged brain. The cause is not fully understood but is has become clear that intrinsic factors in the CNS as well as extrinsic factors in the circulation are contributing factors. They include low level systemic inflammation, increased microglial numbers and activation, declining trophic support, fewer numbers of quiescent neural stem cells, reduced angiogenesis and reduced synaptic plasticity^44,46,47^. Exercise ameliorates neuroinflammation, and enhances neurogenesis, synaptic plasticity, and it increases production and release of neurotropic growth factors^4^, such as BDNF, VEGF, insulin growth factor-1 (IGF-1), fibroblast growth factor-2 (FGF-2), and nerve growth factor (NGF), thus restoring the microenvironment in aging. Exercise also induces PGC-1α and FNDC5 in the muscle and brain, where the PGC-1α pathway is known to regulate mitochondrial biogenesis, oxidative capacity and attenuate ROS production in the CNS^12,35,48^, and as a consequence, mitochondrial biogenesis-dependent recovery^49^ may impact age-dependent decline in hippocampal neurogenesis.

We studied genotype effects on age-dependent reduction in hippocampal cytogenesis and sex-dependent differences as indicated by numbers of newborn cells, immature neurons, and newborn mature neurons at 3 and 11 months of age. However, no differences were observed between genotypes indicating that the MCK-PGC-1α mice do not have altered neurogenesis with aging compared to WT mice. We were unable to detect any differences in cytogenesis in non-neurogenic subregions of the DG. In accordance with this, the volume of the DG for 11-month-old animals was unaffected for both genotypes.

Aging of the brain is characterized by neuroinflammation, a process known to negatively regulate adult hippocampal neurogenesis in rodents and has been found to be present already in middle-age mice^50–52^. The middle-aged mice used in this study show a high lipofuscin load, which indicates that senescent changes, other than a decline in neurogenesis, are also already present in these 11-month-old animals.

Understanding sex difference is of importance for studies of exercise-induced effects on health and disease, including age-related diseases^53^. Female sex hormones have an acute proliferative effect on hippocampal neurogenesis^54^, whereas male sex hormones act positively on cell survival^55^. In this study, we have not controlled for estrogen levels, but found no differences in neurogenesis between sexes neither in young nor in middle-aged mice, which is supported by previous reports^56–58^. However, other studies have found higher levels of proliferation and cell survival in females^59^. Similar to previous reports, we found no differences in total or subregional volume of the DG between the sexes^60^.

The orientation of DCX+ neural progenitor cells in the DG indicates a state of maturity, where parallel orientation (found within the first days after final cell division) and perpendicular orientation (present from about 7 days after cell cycle exit) represents a more immature and a more differentiated cell state, respectively^34^. We did not detect any differences in progenitor maturation between the genotypes in middle-aged females or males.

The irradiated brain shares many similarities with the aged brain, including increased neuroinflammation, reduced numbers of quiescent neural progenitors and lack of trophic support^61^. In our previous study, male MCK-PGC1α mice were not protected against irradiation-reduced neurogenesis (DCX and BrdU/NeuN) in the dentate gyrus 4 weeks after a single dose of cranial irradiation (4 Gy) at 4 months of age^31^. This confirms our findings that chronic upregulation of muscular PGC-1α does not lead to reduced neuroinflammation, increased stem cell activation or increased trophic support. Together these findings indicate that muscular overexpression of PGC-1α does not affect neurogenesis or inflammatory response with aging.

Levels of cytokines, chemokines, and myokines were analysed in serum of 11-month-old wildtype and transgenic animals, using commercially available multiplex kits. We observed a tendency towards increased levels of myokines and reduced levels of pro-inflammatory cytokines in transgenic animals, but only the myokine musclin was significantly upregulated in transgenic animals. Musclin, also known as osteocrin, is an exercise-induced myokine essential for increased endurance capacity through mitochondrial biogenesis and muscle fiber-type switching^62^. Due to the shared structural homology with the cardiac naturetic peptide that has been described to not only have beneficial effects on vasculature and kidneys, but also induce mitochondrial biogenesis, angiogenesis, lipolysis, adipocyte tissue remodeling, it is possible that the musclin is involved in systemic effects on other tissues as well^62^. Musclin has been identified as a activity-dependent secreted factor in human fetal brain cultures (but not mouse neuronal cultures) binding to MEF2, and is implicated in human cognition where it has been reported to restrict activity-dependent dendritic growth in human neurons^63^. With regards to systemic changes in MCK-PGC-1α mice a recent study by Peng and colleagues reported that the transgenic mice had improved kidney energy metabolism with protection from tubular damage^23^. This effect was found to be mediated by a low molecular weight fraction of serum in MCK-PGC-1α mice, reported to be dependent on irisin signaling. The authors reported a 3-4-fold upregulation in muscular gene expression of *Fndc5*, *Bdnf*, and *Il15*, among other genes, and a 2-fold increase in serum levels of irisin, BDNF, and IL-15, in MCK-PGC-1α animals.

VEGF is a known peripheral mediator of neurogenesis and has been reported to be essential for exercise-induced neurogenesis^38^. We observe an upregulation of *Vegfb* in skeletal muscle of MCK-PGC-1α mice, along with other genes with neurotrophic potential, without differences in basal levels of hippocampal neurogenesis. In accordance with our findings, Agudelo and colleagues reported that gene expression of the growth factors *Bdnf*, *Gdnf*, *Ngf*, *Vegfa*, and *Vegfb*, were unaltered in the hippocampus of MCK-PGC-1α mice^24^. Acute overexpression of PGC-1α has been demonstrated to upregulate short-term VEGF protein expression in skeletal muscle cells^64^. However, chronic overexpression of PGC-1α may, through compensatory adaptations, result in unchanged VEGF protein levels. Considering that the half-life of VEGF in serum has been reported to be very short in mouse (approximately 3 minutes)^65^, the high clearance rate would effectively prevent accumulation in the circulation from a steady-state expression in skeletal muscle. As mentioned above, Peng and colleagues reported that gene expression of *Fndc5*, *Bdnf*, *Il15*, *Angptl4*, *Fgf21*, and *Ctsb*, but not *Vegf*, were upregulated in skeletal muscle of MCK-PGC-1α mice, with elevated serum levels of irisin, BDNF, and IL-15. Despite this report of systemically upregulated neurotrophic factors in serum of these animals, we do not detect a difference in neurogenesis in our transgenic mouse model. It is possible that compensatory adaptations occurring over time from chronically elevated systemic myokines leads to steady-state effects on hippocampal neurogenesis and hippocampal gene expression.

In this paper, we focused on analysing hippocampal neurogenesis since it is a prominent phenomenon of exercise-inducible effects on the brain. It should be noted that we did not investigate potential differences in in other types of neural plasticity or behavior. Also, compensatory adaptations in molecular pathways due to the constitutive overexpression of PGC-1α during development could be present. Exercise has a plethora of effects in the body and the brain by activating a complex network of pathways in different cell types, tissues and organs. Genetic and pharmacological approaches to muscle activation have not yet been reported to yield sustainable effects. There is an apparent link between muscle and brain during exercise, but the time-dependent dynamic aspects of this signaling needs to be studied further. We demonstrate that a sustained upregulation of the muscular PGC-1α pathway, despite potent systemic changes, does not protect from age-dependent decline of hippocampal neurogenesis. We conclude that, at least with regard to aging, PGC-1α overexpression is not sufficient to mimic exercise-induced effects on the brain. Continued studies will elucidate the complex molecular mechanisms governing rejuvenating effects of exercise on the CNS in disease and aging, with hope to discovering new therapeutic possibilities for patients.

## Supplementary information

Supplementary Material

## Data Availability

The datasets generated during and/or analysed during the current study are available from the corresponding author on reasonable request.
