# Supplementary material for: Constitutive PGC-1α overexpression in skeletal muscle does not protect from age-dependent decline in neurogenesis
Source: Sci Rep. 2019 Aug 23;9:12320. doi: 10.1038/s41598-019-48795-w (PMC6707251; doi:10.1038/s41598-019-48795-w)
Supplement: Supplementary file 1 — Supplementary Material [file 41598_2019_48795_MOESM1_ESM.docx]

# SUPPLEMENTARY MATERIAL

Title: Constitutive PGC-1α overexpression in skeletal muscle does not protect from age-dependent decline in neurogenesis

Author List: Lars Karlsson*, González-Alvarado María Nazareth, Reza Motalleb, Klas Blomgren, Mats Börjesson, Hans Georg Kuhn

**Supplementary Table S1.** List of qPCR primers.

| **Symbol** | **Name** | **Forward primer sequence** | **Reverse primer sequence** |
| --- | --- | --- | --- |
| *18S* | 18S ribosomal RNA | TCAAGAACGAAAGTCGGAGG | GGACATCTAAGGGCATCAC |
| *Actb* | Beta-actin | CACGATGGAGGGGAATACAGC | GCCTTCTTTTGTGTCTTGATAGTTC |
| *Bdnf* | Brain-derived neurotrophic factor | GGCTGACACTTTTGAGCACG | CAAGTCCGCGTCCTTATGGT |
| *Ctsb* | Cathepsin B | AGACCTGCTTACTTGCTGTG | GGAGGGATGGTGTATGGTAAG |
| *Fndc5* | Fibronectin type III domain-containing protein 5 | GGACTCTTGGAAAACACCACTG | TCCACACAGATGATCTCACCAC |
| *Gapdh* | Glyceraldehyde 3-phosphate dehydrogenase | CCTCATGGGTCTGTAGTGAGC | TTGTGGTACGTGCATAGCTG |
| *Il15* | Interleukin 15 | GAGGCCAAGAAGAGTTCTGGA | TGCCCAGGTAAGAGCTTCAA |
| *Timp4* | Metalloproteinase inhibitor 4 | GACCCTGCTGACACTCAAAAAC | GGAAGAGTCAAATGGCGTGTAG |
| *Pgc1a* | Peroxisome proliferator-activated receptor gamma coactivator 1 | CCCTGCCATTGTTAAGACC | TGCTGCTGTTCCTGTTTTC |
| *Tbp* | TATA-binding protein | GAAGCTGCGGTACAATTCCAG | CCCCTTGTACCCTTCACCAAT |
| *Vegfb* | Vascular endothelial growth factor B | TATCTCCCAGAGCTGCCATCTA | AGCCAGAAGATGCTCACTTGAC |

**Supplementary Table S2.** Main and interaction effects of two-way ANOVA on subregional volumes of the DG and corresponding number of newborn cells in 11-month-old animals.

| Analysis^a^  GCL volume^b^  ML volume^b^  Hilus volume^b^  DG BrdU^+^ cells^c^  ML BrdU^+^ cells^c^  Hilus BrdU^+^ cells^c^ | Genotype effect  1.19 (p=0.28)  1.33 (p=0.26)  0.001 (p=0.97)  0.045 (p=0.83)  0.084 (p=0.77)  5.39 (p=0.026)* | Sex effect  2.49 (p=0.12)  4.58 (p=0.04)*  2.94 (p=0.096)  0.11 (p=0.74)  0.36 (p=0.55)  0.77 (p=0.39) | Interaction effect  2.00 (p=0.17)  2.94 (p=0.09)  1.01 (p=0.32)  0.078 (p=0.78)  0.010 (p=0.92)  3.74 (p=0.06) |
| --- | --- | --- | --- |

^a^ See Fig. 2A-F.

^b^ Data presented as test value of F(1, 30) along with the corresponding p-value in parenthesis.

^c^ Data presented as test value of F(1, 39) along with the corresponding p-value in parenthesis.

**Supplementary Table S3.** Main and interaction effects of two-way ANOVA on number of immature (parallel) and mature (perpendicular) DCX^+^ cells in the DG.

| Analysis^a^  DCX^+^ cells parallel^b^  DCX^+^ cells perpendicular^b^ | Genotype effect  0.016 (p=0.90)  0.013 (p=0.91) | Sex effect  1.38 (p=0.25)  0.62 (p=0.44) | Interaction effect  0.15 (p=0.71)  1.60 (p=0.22) |
| --- | --- | --- | --- |

^a^ See Fig. 4.

^b^ Data presented as test value of F(1, 30) along with the corresponding p-value in parenthesis.

**Supplementary Table S4.** Parametric and non-parametric statistics on serum concentrations of cytokines, chemokines, and myokines.

| Analysis^a^   \| BDNF^d^ \| \| --- \| \| FGF21^c^ \| \| Myostatin^d^ \| \| Osteocrin^d^ \| \| Osteonectin^c^ \| \| ENA-78^b,c^ \| \| Eotaxin^b,c^ \| \| G-CSF^d^ \| \| GRO-alpha^c^ \| \| IFN-gamma^d^ \| \| IL-1alpha^c^ \| \| IL-1beta^d^ \| \| IL-10^c^ \| \| IL-18^c^ \| \| IL-22^c^ \| \| IL-23^b,c^ \| \| IL-27^c^ \| \| IL-28^d^ \| \| IL-4^c^ \| \| IL-5^d^ \| \| IL-6^d^ \| \| IL-9^d^ \| \| IP-10^d^ \| \| LIF^d^ \| \| MCP-1^c^ \| \| MCP-3^c^ \| \| MIP-1beta^c^ \| \| MIP-2^c^ \| \| RANTES^c^ \| | P-value   \| 0.34 \| \| --- \| \| 0.30 \| \| 0.18 \| \| 0.0029 \| \| 0.53 \| \| 0.39 \| \| 0.18 \| \| 0.67 \| \| 0.20 \| \| 0.38 \| \| 0.33 \| \| 0.35 \| \| 0.16 \| \| 0.40 \| \| 0.24 \| \| 0.98 \| \| 0.36 \| \| 0.69 \| \| 0.10 \| \| 0.16 \| \| 0.22 \| \| 0.74 \| \| 0.32 \| \| 0.11 \| \| 0.04 \| \| 0.01 \| \| 0.08 \| \| 0.90 \| \| 0.32 \| | FDR-adjusted p-value   \| 0.55 \| \| --- \| \| 0.62 \| \| 0.58 \| \| 0.084 \| \| 0.64 \| \| 0.51 \| \| 0.52 \| \| 0.78 \| \| 0.53 \| \| 0.52 \| \| 0.56 \| \| 0.53 \| \| 0.66 \| \| 0.50 \| \| 0.54 \| \| 0.98 \| \| 0.52 \| \| 0.77 \| \| 0.58 \| \| 0.58 \| \| 0.53 \| \| 0.79 \| \| 0.62 \| \| 0.53 \| \| 0.39 \| \| 0.15 \| \| 0.58 \| \| 0.93 \| \| 0.58 \| |
| --- | --- | --- | --- | --- | --- | --- | --- | --- | --- | --- | --- | --- | --- | --- | --- | --- | --- | --- | --- | --- | --- | --- | --- | --- | --- | --- | --- | --- | --- | --- | --- | --- | --- | --- | --- | --- | --- | --- | --- | --- | --- | --- | --- | --- | --- | --- | --- | --- | --- | --- | --- | --- | --- | --- | --- | --- | --- | --- | --- | --- | --- | --- | --- | --- | --- | --- | --- | --- | --- | --- | --- | --- | --- | --- | --- | --- | --- | --- | --- | --- | --- | --- | --- | --- | --- | --- | --- | --- | --- |

^a^ See Fig 5.
^b^ Log transformed data. ^c^ t-test, n=7-10.
^d^ Mann-Whitney, n=7-10.
